# Sex differences in secondary preventive follow-up after coronary heart events

**DOI:** 10.1186/s12872-023-03483-6

**Published:** 2023-09-14

**Authors:** Anete Kaldal, Serena Tonstad, Jarle Jortveit

**Affiliations:** 1 Department of Research, Sørlandet Hospital, Kristiansand S, Norway; 2 Department of Endocrinology, Obesity and Preventive Medicine, Section of Preventive Cardiology, Oslo University Hospital, Oslo, Norway; 3 Department of Cardiology, Sorlandet Hospital, Arendal, Norway

**Keywords:** Sex differences, Cardiovascular diseases, Secondary prevention

## Abstract

**Background and aims:**

Some studies point to sex differences in cardiovascular preventive practices. The aim of this study was to investigate differences in achievement of secondary preventive targets and long-term outcome in men and women after a coronary heart event.

**Methods:**

This study was a subanalysis from a randomized controlled trial of hospital-based versus primary care-based secondary preventive follow-up at Sorlandet Hospital, Norway, 2007–2022 and included both groups. The main outcome was achievement of treatment targets two years after the index event. Event-free survival was calculated based on the composite of mortality, coronary intervention, stroke, or myocardial infarction during follow-up. Participants were followed-up for up to 10 years after the index event through out-patient consultations.

**Results:**

In total, 337 women and 1203 men were eligible for the study. Due to loss of follow-up during the first two years after the index coronary event 106 (7%) participants were excluded from further analysis (53% withdrawal of consent, 12% death, and 35% other causes) leaving 307 (21%) women and 1127 (79%) men. After two years of follow-up we found no differences between women and men in achievement of blood pressure targets (61% vs. 59%; *p* = 0.57), LDL-cholesterol goals (64% vs. 69%; *p* = 0.15), HbA1c-goal in patients with diabetes (49% vs. 45%; *p* = 0.57), non-smoking (79% vs. 81%; *p* = 0.34), healthy diets (14% vs. 13%, *p* = 0.89), physical activity (55% vs. 58%; *p* = 0.38), use of acetylsalicylic acid (93% vs. 94%; *p* = 0.39), and use of lipid lowering therapy (92% vs. 94%; *p* = 0.15). After a median follow-up time of 5.0 [SD 3.2] years there were no differences between women and men regarding composite endpoint (89 [30.0%] vs. 345 [30.6]; *p* = 0.58), and composite endpoint-free survival did not differ between women and men (hospital-based follow-up HR for women versus men, 0.87, 95% CI 0.62–1.23; *p* = 0.44 and primary care service HR for women versus men 0.95, 95% CI 0.69–1.31; *p* = 0.78).

**Conclusions:**

The study show no sex differences in achievement of secondary preventive targets or composite endpoint after coronary heart events. However, many women and men did not achieve treatment goals, and further improvement in secondary prevention is needed.

**Trial registration:**

The study is registered in ClinicalTrials.gov (NCT00679237).

**Supplementary Information:**

The online version contains supplementary material available at 10.1186/s12872-023-03483-6.

## Introduction

Cardiovascular disease (CVD) is still a leading cause of death in Europe, accounting for 45% of women’s and 39% of men’s deaths [1]. Many patients with CVD experience repeated CVD events [2]. The European Society of Cardiology (ESC) and American Heart Association (AHA)/American College of Cardiology (ACC) have issued detailed guidelines on primary and secondary prevention of CVD [3, 4]. However, large studies have demonstrated a remaining gap between the guidelines and the achievement of recommended targets [5–8]. Furthermore, cardiovascular mortality rates are higher in women than in men [9–13]. Several studies have revealed that women may have less clear symptoms and clinical findings of acute myocardial infarction (MI), wait longer for treatment, less frequently undergo invasive assessment and were prescribed less secondary prophylactic medication at hospital discharge after acute MI compared to men [13–24]. Some registry studies have indicated lower risk factor assessment for secondary prevention for women than men, especially in the primary care setting, but whether the difference in acute treatment is reflected in poorer secondary preventive treatment is still unclear [25, 26].

The aim of the present subanalysis from a prospective randomized controlled trial of hospital-based versus primary care-based follow-up was to investigate sex differences in achievement of secondary preventive targets after MI, percutaneous coronary intervention (PCI) and coronary artery bypass grafting (CABG).

## Methods

### Study design and study population

The study was conducted as a subanalysis from a randomized controlled trial at Sorlandet Hospital Arendal, Norway in the period 2007–2022. The main study focused on differences between secondary cardiovascular prevention within primary health care and within hospital-based follow-up, and primary outcomes were all-cause mortality and composite endpoint of all-cause mortality, recurrence of non-fatal MI, new PCI/CABG, and non-fatal stroke.

Consecutive women and men, aged 18 to 80 years, admitted to the hospital with a diagnosis of MI or after scheduled PCI/CABG were randomized to hospital-based follow-up or to follow-up within the primary health care [27]. The exclusion criteria were as follows: lack of ability to cooperate, known alcohol- or drug-abuse, use of narcotics, pregnancy or breast-feeding, serious comorbidity with a life expectancy less than two years, or participation in other secondary prevention studies [27]. The main study is described in detail previously [27].

### Intervention (hospital-based follow-up)

Regular outpatient consultations were offered for patients in the hospital-based follow-up group. Specially trained nurses, supervised by cardiologists, followed up patients starting from the first consultation during the hospital admission for the index event, at two weeks, three months, six months and thereafter annually for up to five years after the index event, with final data collection after 10 years [27]. The attainment of treatment targets was evaluated at each consultation, and following measures were assessed: blood pressure, weight, height, waist circumference, LDL-cholesterol and HbA_1c_. Smoking status, diet, physical activity, and use of medication were reported by the patient. At each consecutive consultation data about death, hospital admissions, stroke, recurrent MI, or new PCI/CABG were recorded [27].

#### Intervention measures

- Smoking cessation: Nicotine replacement therapy (NRT) was offered during hospital admission, and continuation of NRT or initiation of a 12-week course of varenicline after discharge was advised.
- Blood pressure: In addition to the promotion of a healthy lifestyle, pharmacological antihypertensive therapy was initiated and/or adjusted. The choice of medication was based on an individual clinical evaluation of each patient.
- All participants were prescribed statins unless contraindicated, and other lipid lowering agents (primarily ezetimibe) were added to treatment if statins alone did not provide recommended result.
- Patients with a diagnosis of diabetes mellitus were identified and antidiabetic therapy was initiated and/or adjusted after clinical evaluation.
- Physical activity of moderate intensity ≥ 150 min weekly was advised to all participants.
- SmartDiet^tm^ [28] scoring was used to assess dietary habits. Individual nutritional guidance was provided based on the responses.
- Acetylsalicylic acid (ASA) was prescribed accordingly to clinical guidelines [27].

In the primary care group, the secondary preventive follow-up was conducted by the family physician. Advice regarding treatment targets was sent to the family physician when the patient was discharged from hospital after the index event. Study data in the primary care group was obtained through regular outpatient consultations at 12 months, two years, and five years with a final data collection at ten years after the index event [27]. The study data collected through outpatient consultations was identical as in the hospital-based follow-up group, but without intervening in the treatment regimes [27].

#### Treatment targets of secondary prevention

The secondary preventive treatment targets adhered to the latest ESC guidelines available [4, 27, 29–35].

- No smoking
- Blood pressure < 140/90 mmHg
- LDL-cholesterol < 1.8 mmol/l (< 2.5 mmol/l until 2017, < 1.4 mmol/l from 2020)
- HbA_1c_ < 53 mmol/mol (7%)
- BMI < 25 kg/m^2^
- Healthy diet (defined as SmartDiet.^tm^ score ≥ 36 points)
- Daily use of statins
- Daily use of acetylsalicylic acid
- Physical activity of moderate intensity ≥ 150 min weekly

### Outcomes

The main outcome for the subanalysis was achievement of treatment targets of secondary prevention for cardiovascular risk factors and medication use two years after the index coronary heart event among men and women in total, and in each study group. The two-year follow-up was considered optimal for analysis as it would provide relatively long follow-up period while avoiding weakened analysis due to increasing loss to follow-up over the study period. In addition, an event-free survival was calculated based on composite of all-cause mortality, PCI, CABG, non-fatal stroke, or non-fatal MI (first event) during the follow-up for both sexes.

### Statistical analysis

Continuous variables are reported as means with ± SD (standard deviations), and for analyzing differences between groups independent samples t-tests were used. For categorical variables numbers and percentages were used to present data, and the chi-squared test applied to analyze differences between groups. Missing values are presented, and for categorical variables proportion of non-missing values are reported. Kaplan–Meier curve for composite endpoint-free survival after the index event (MI or PCI/CABG) in the study period was estimated for both sexes in either of study groups. Hazard ratios (HRs) with 95% confidence intervals (CIs) were calculated by Cox regression. A *p*-value of < 0.05 was regarded as statistically significant. The analysis was not adjusted for multiple testing. The statistical analyses were carried out by STATA, version 17 (StataCorp, 4905 Lakeway Dr, College Station, TX 77845, USA).

## Results

**Fig. 1:**
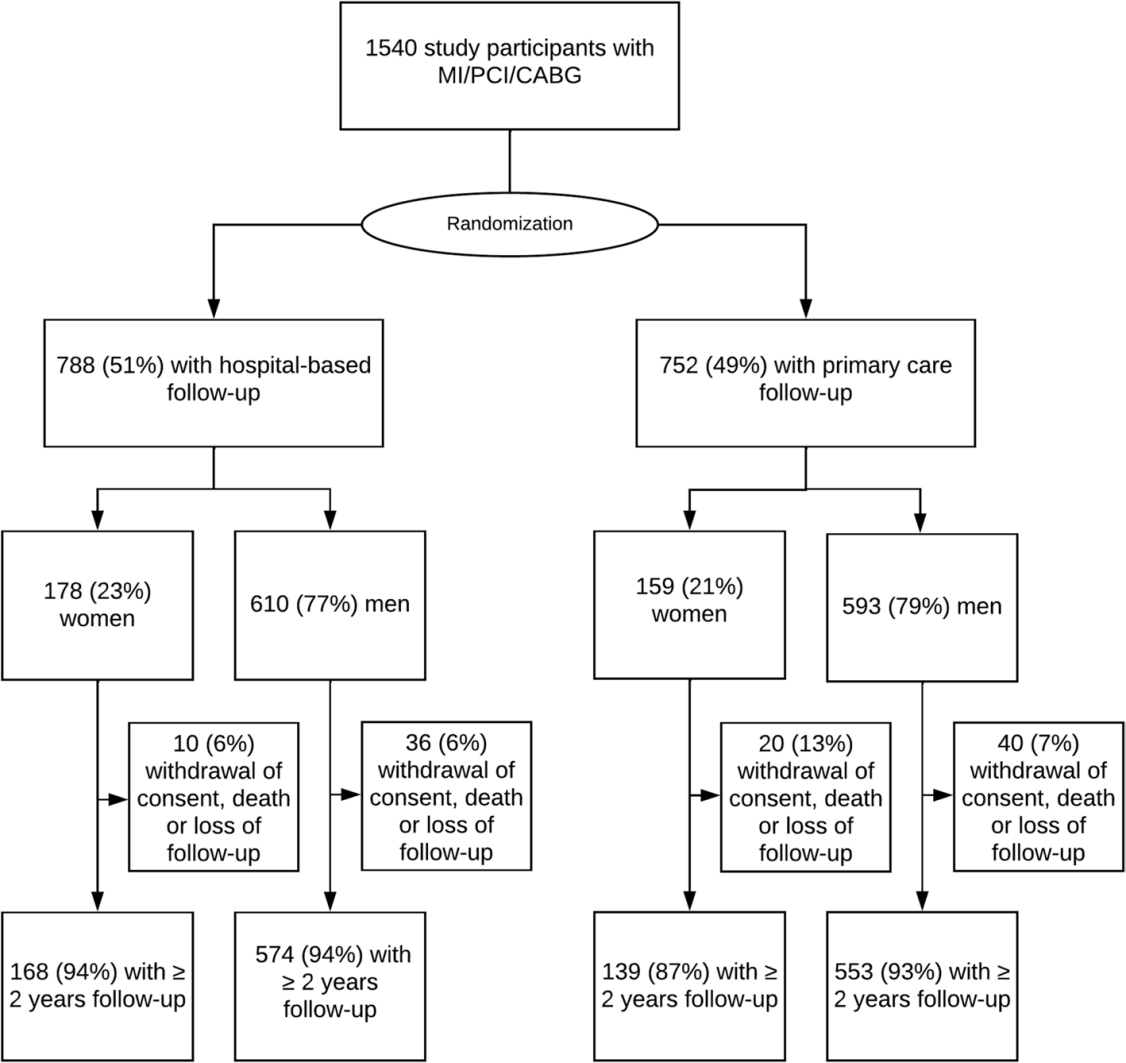
Study flow chart

A total of 1540 patients were included in the main study during the inclusion period from 2007 to 2017. Due to loss of follow-up during the first two years after the index coronary heart event 106 (7%) patients were excluded from further analysis (53% withdrawal of consent, 12% death, and 35% other causes) (Fig. 1). More women than man (13% vs. 7%, *p* = 0.02) chose to discontinue participation in the study during the first two years in the primary care group.

### Clinical characteristics

Baseline characteristics of all the 1434 patients included in this subanalysis are described in Table 1. Baseline characteristics separately for the 742 (52%) participants randomized to hospital-based follow-up and for the 692 (48%) participants with follow-up in the primary care service are described in Supplemental Table 1. Half of the patients (*n* = 693 (49%)) were hospitalized due to acute MI and the others were included in the study after scheduled PCI or CABG. A total of 168 (23%) and 139 (20%) of the patients were women in the groups with hospital-based and primary care follow-up, respectively. The women were older and had a lower BMI than the men. Greater proportion of women received antihypertensive treatment and had higher systolic blood pressure, while more men had previous coronary heart disease.

**Table 1:** Baseline clinical characteristics at hospitalization for index event in women and men with hospital-based secondary preventive follow-up program or primary care-based follow-up after acute myocardial infarction (MI), percutaneous coronary intervention (PCI) or coronary artery bypass grafting (CABG)

		**Women**	**Men**	***p***
*n* = 1434	Missing values			
		*n *= 307	*n* = 1127	
Mean age (years) (SD)	0 (0)	65 (9)	63 (9)	< 0.001
Higher education (%)	78 (5)	58 (20)	325 (30)	0.001
Working (%)	11 (1)	80 (26)	474 (42)	< 0.001
Married/cohabiting (%)	0 (0)	219 (71)	937 (83)	< 0.001
Mean body mass index (kg/m2) (SD)	146 (10)	27.6 (5.0)	28.2 (4.1)	0.04
Smoking (%)	5 (0)	86 (28)	294 (26)	0.47
Lipid lowering therapy (%)	39 (3)	133 (44)	506 (46)	0.38
Antihypertensive therapy (%)	23 (2)	170 (56)	508 (46)	0.002
Diabetes (%)	4 (0)	56 (18)	167 (15)	0.14
Previous coronary heart disease				
Myocardial infarction (%)	6 (0)	25 (8)	168 (15)	0.002
Percutaneous coronary intervention (%)	2 (0)	29 (9)	165 (15)	0.02
Coronary artery bypass grafting (%)	2 (0)	7 (2)	75 (7)	0.003
Previous stroke (%)	7 (0)	19 (6)	50 (4)	0.20
Mean LDL-cholesterol (mmol/L) (SD)	47 (3)	3.0 (1.1)	2.9 (1.1)	0.52
Mean systolic blood pressure (mmHg) (SD)	2 (0)	149 (27)	146 (23)	0.05
Mean diastolic blood pressure (mmHg) (SD)	2 (0)	86 (15)	87 (14)	0.45
Mean left ventricular ejection fraction (%) (SD)	322 (22)	52 (11)	52 (11)	0.56
Acute myocardial infarction (%)	25 (2)	150 (50)	543 (49)	0.80

### Outcomes

Achievement of treatment targets for secondary prevention after two years are presented in Table 2 and Supplemental Table 2. We found no significant differences between women and men regarding secondary preventive target achievement for cardiovascular risk factors and use of secondary preventive medication after two years, except for BMI and waist circumference. More patients with hospital-based follow-up reached treatment goals for blood pressure, LDL cholesterol, healthy diet and physical activity compared to patients with primary care follow-up, but there were no major sex differences within the groups.

**Table 2:** Secondary preventive target achievement for cardiovascular risk factors and medication use in women and men two years after acute myocardial infarction (MI), percutaneous coronary intervention (PCI) or coronary artery bypass grafting (CABG)

**Target achievement, (%)**^a^	Missing values	**Women**	**Men**	***p***
*n* = 1434		*n* = 307	*n* = 1127	
Blood pressure	2 (0)	187 (61)	668 (59)	0.57
LDL-cholesterol	24 (2)	194 (64)	763 (69)	0.15
HbA1c (if diabetes, *n* = 240)	11 (1)	28 (49)	77 (45)	0.57
Body mass index	6 (0)	106 (35)	241 (21)	< 0.001
Waist circumference	12 (1)	35 (12)	262 (23)	< 0.001
Lipid lowering therapy	13 (1)	280 (92)	1054 (94)	0.15
Acetylsalicylic acid	3 (0)	284 (93)	1055 (94)	0.39
Healthy diet	13 (1)	41 (14)	148 (13)	0.89
Non-smoking	0 (0)	241 (79)	912 (81)	0.34
Physical activity	0 (0)	168 (55)	648 (58)	0.38

^a^Blood pressure < 140/90 mmHg, LDL-cholesterol < 2.5 mmol/l (until 2017)/ < 1.8 mmol/l (2018–2020)/ < 1.4 mmol/l (2021-), HbA1c < 53 mmol/l (7%), body mass index < 25 kg/m2, waist circumference < 80 cm (women)/ < 94 cm (men), daily use of lipid lowering therapy, daily use of acetylsalisylic acid, Smart Diet score ≥ 36, non-smoking, physical activity of minimum moderate intensity ≥ 150 min weekly

A total of 30 (35%) women and 105 (36%) men (*p* = 0.89) who smoked at inclusion quitted within two years after the index event. We found no differences between hospital- and primary care-based follow-up.

**Fig. 2:**
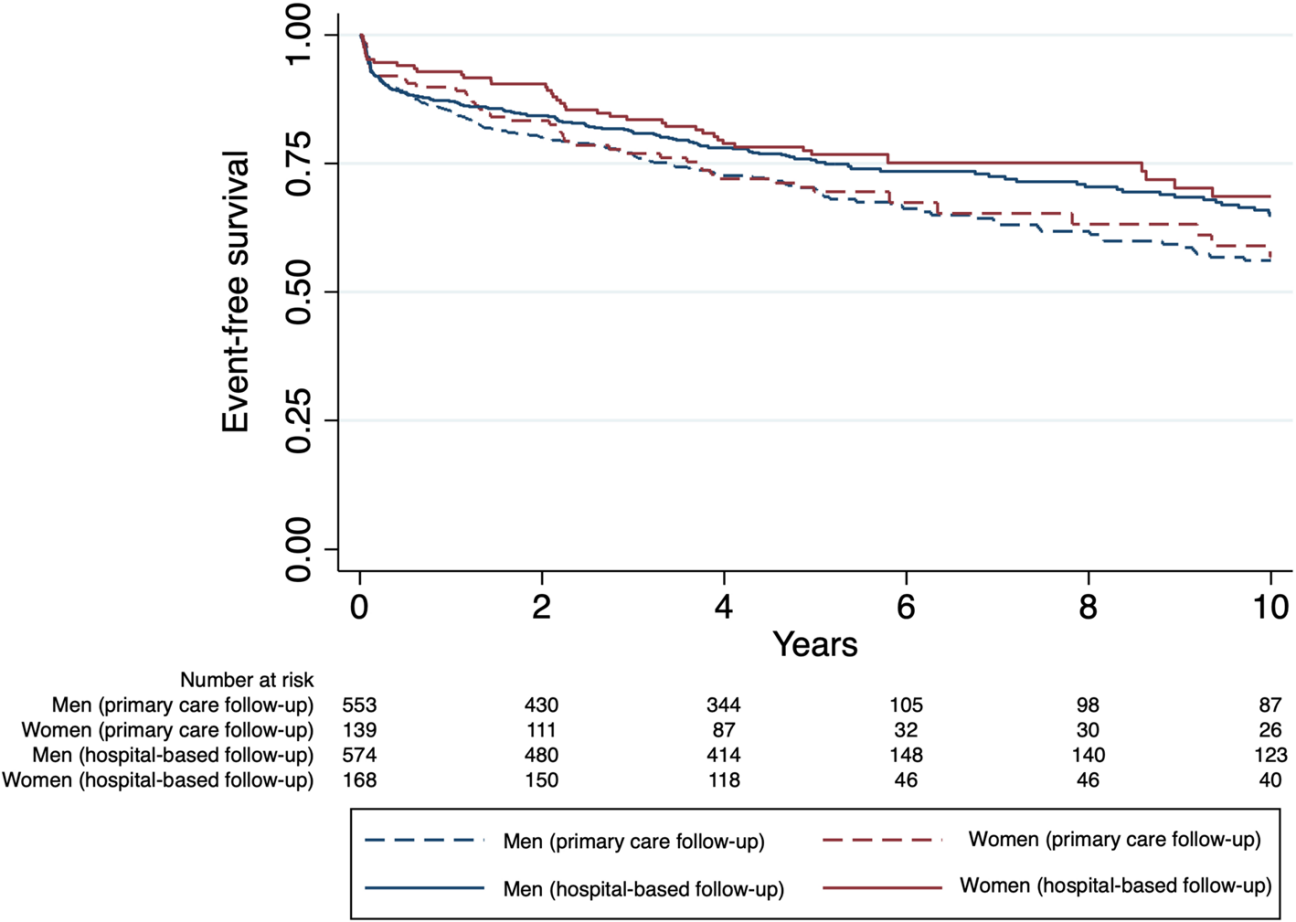
Composite endpoint-free survival in women and men with hospital- and primary care-based secondary preventive follow-up program after myocardial infarction (MI), percutaneous coronary intervention (PCI) or coronary artery bypass grafting (CABG)

After a median follow-up time of 5.0 (SD 3.2) years there were no sex differences in composite endpoint-free survival neither in patients with hospital-based follow-up (HR women 0.87, 95% CI 0.62–1.23, *p* = 0.44) nor in patients with follow-up in the primary care service (HR women 0.95, 95% CI 0.69–1.31, *p* = 0.78) (Table 3 and Fig. 2). However, more patients in the primary care follow-up group underwent a new PCI procedure (HR 1.43, 95% CI 0.14–1.79, *p* = 0.002) compared to the hospital-based follow-up group.

**Table 3:** Composite endpoint (all-cause death, non-fatal myocardial infarction (MI), percutaneous coronary intervention (PCI), coronary artery bypass grafting (CABG) or non-fatal stroke) and total number of cardiovascular events in women and men with hospital-based or primary care-based secondary preventive follow-up after acute myocardial infarction (MI), percutaneous coronary intervention (PCI) or coronary artery bypass grafting (CABG)

	**Women**				**Men**				***p***		
	All	Hospital-based follow-up	Primary care-based follow-up	*p*	All	Hospital-based follow-up	Primary care-based follow-up	*p*		p*	p**
	*n* = 307	*n* = 168	*n* = 139		*n* = 1127	*n* = 574	*n* = 553				
**Composite endpoint**	89 (30.0)	42 (25.0)	37 (33.8)	0.09	345 (30.6)	161 (28.1)	184 (33.2)	0.06	0.58	0.44	0.90
Death	9 (2.9)	5 (3.0)	4 (2.9)	0.96	46 (4.1)	22 (3.8)	24 (4.3)	0.67	0.35	0.60	0.43
Myocardial infarction	17 (5.5	10 (6.0)	7 (5.0)	0.73	60 (5.3)	28 (4.9)	32 (5.8)	0.50	0.88	0.59	0.73
Percutaneous coronary intervention	69 (22.5)	30 (17.9)	39 (28.1)	0.03	247 (21.9)	110 (19.2)	137 (24.8)	0.02	0.83	0.70	0.43
Coronary artery bypass grafting	0 (0)	0	0	NA	21 (1.9)	9 (1.6)	12 (2.2)	0.46	0.02	0.1	0.08
Stroke	15 (4.9)	9 (5.4)	6 (4.4)	0.70	51 (4.6)	26 (4.6)	25 (4.6)	1	0.78	0.66	0.93

Mean follow-up time was 5.0 (SD 3.2) years

^*^Hospital-based follow-up: women vs. men

^**^Primary care-based follow-up: women vs. men

## Discussion

This subanalysis from a randomized controlled trial showed no significant sex differences in secondary preventive target achievement for cardiovascular risk factors and medication use or for composite endpoint-free survival after MI, PCI or CABG. We found no sex differences between patients with hospital-based follow-up and patients with follow-up in the primary care service.

Sex differences in presentation and in the management of acute coronary heart syndrome are well-described, but not well-understood [13, 19, 24, 36–38]. International guidelines do not distinguish between women and men in their recommendations for treatment of coronary heart disease. However, women and men with MI may have different risk factors, coronary artery anatomy, and comorbidities. The underlying mechanisms of MI may differ in women and men. Inflammation, endothelial dysfunction, connective tissue disorders, coronary vasospasm, spontaneous coronary artery dissection may play a more important role in women [15, 37, 39, 40]. Greater comorbidity in women may also help to explain some of the sex differences in hospital treatment.

Hyun et al. and Lee et al. have described less use of secondary preventive medication and higher incidence of major adverse cardiovascular events after acute coronary syndrome in Australian women compared to their male counterparts [25, 26]. These sex differences were not confirmed in our study. However, this study revealed a potential for improvement in the overall rate of treatment target achievement for both women and men. 20–40% of the participants with hospital-based follow-up did not reach the treatment target for blood-pressure and LDL-cholesterol. The adherence to guidelines was even lower in the primary care follow-up group. The majority of the patients did not achieve treatment targets for the lifestyle factors body mass index, healthy diet, and physical activity. Only one in three smokers quit smoking. For the lifestyle factors, this study revealed no differences between hospital and primary care follow-up. We find reason to stress the importance of adherence to the secondary preventive guidelines from ESC, AHA and ACC for all patients – both women and men and both in hospital and primary care settings.

About 30% of all participants experienced a new major cardiovascular event (MACE) during the follow-up period (mean 5 years). This reiterates the importance of improving secondary preventive treatment after MI, PCI and/or CABG.

The main strengths of this study are the inclusion of both patients with hospital-based and primary care-based follow-up and the low proportion of dropouts. Selection bias due to socioeconomical status is supposed to be minimal, given that patient charges in Norway are low and availability of healthcare services is independent of income level. Whether educational or other factors contribute to some degree of selection bias, require an analysis of patient characteristics among those who refused participation in the study, which was not available for us. The effects of healthcare provider-patient gender concordance/discordance on the treatment choices and results has been questioned in several studies, but the data is scarce and results far from conclusive and unambiguous [41]. We have not specifically examined possible gender concordance or discordance effects as there were only female nurses involved in our study, and the treatment goals were purely determined by the current guidelines, although allowing individual approach in the choice of medical therapy. This study is limited to one hospital and a limited number of participants. Generalization of the findings must therefore be done with great caution. Smoking status, dietary habits, amount of exercise and use of medications were self-reported, and likely to be affected by reporting bias.

The study results may be influenced as well by smaller proportion of women than men participating in the study. We assume that the open design of the study implies awareness of participation, which might have influenced the behavior of participants. We also lacked an overview of the number of consultations and treatment measures at the primary care service.

## Conclusion

Although this study did not show any sex differences in achievement of treatment targets for secondary prevention and outcome after a coronary heart event, many patients did not achieve the secondary preventive treatment targets and experienced a new major cardiovascular event within few years. Further efforts should be made to improve the treatment of men and women with coronary heart disease.

### Supplementary Information

**Additional file 1: Supplemental table 1.** Baseline clinical characteristics at hospitalization for index event in women and men with hospital-based and primary care-based secondary preventive follow-up program after acute myocardial infarction (MI), percutaneous coronary intervention (PCI) or coronary artery bypass grafting (CABG). **Supplemental table 2.** Secondary preventive target achievement for cardiovascular risk factors and medication use in women and men two years after acute myocardial infarction (MI), percutaneous coronary intervention (PCI) or coronary artery bypass grafting (CABG).

## Data Availability

The study protocol and data are available on request due to privacy/ethical restrictions, the corresponding author should be contacted in case.

## Abbreviations:

BMI: Body mass index
CABG: Coronary artery bypass grafting
CVD: Cardiovascular diseases
HbA_1c_: Glycated heamoglobin
LDL: Low-density lipoprotein
MI: Myocardial infarction
PCI: Percutaneous coronary intervention
