# Supplementary material for: Sex differences in secondary preventive follow-up after coronary heart events
Source: BMC Cardiovasc Disord. 2023 Sep 14;23:459. doi: 10.1186/s12872-023-03483-6 (PMC10502978; doi:10.1186/s12872-023-03483-6)
Supplement: Supplementary file 1 — Additional file 1: Supplemental table 1. Baseline clinical characteristics at hospitalization for index event in women and men with hospital-based and primary care-based secondary preventive follow-up program after acute myocardial infarction (MI), percutaneous coronary intervention (PCI) or coronary artery bypass grafting (CABG). Supplemental table 2. Secondary preventive target achievement for cardiovascular risk factors and medication use in women and men two years after acute myocardial infarction (MI), percutaneous coronary intervention (PCI) or coronary artery bypass grafting (CABG). [file 12872_2023_3483_MOESM1_ESM.docx]

**Supplemental tables**

| **Supplemental table 1. Baseline clinical characteristics at hospitalization for index event in women and men with hospital-based and primary care-based secondary preventive follow-up program after acute myocardial infarction (MI), percutaneous coronary intervention (PCI) or coronary artery bypass grafting (CABG).** | | | | | | | | |
| --- | --- | --- | --- | --- | --- | --- | --- | --- |
|  | **Women** | | | **Men** | | |  |  |
|  | Hospital-based follow-up | Primary care-based follow-up | *p* | Hospital-based follow-up | Primary care-based follow-up | *p* | *p** | *p*** |
| n=1434 | n=168 | n=139 |  | n=574 | n=553 |  |  |  |
| Mean age (years) (SD) | 64 (9) | 65 (9) | 0.20 | 62 (10) | 64 (9) | <0.001 | 0.004 | 0.05 |
| Higher education (%) | 31 (20) | 27 (20) | 0.95 | 161 (29) | 164 (329) | 0.38 | 0.03 | 0.01 |
| Working (%) | 46 (27) | 34 (25) | 0.59 | 248 (44) | 226 (41) | 0.43 | <0.001 | <0.001 |
| Married/cohabiting (%) | 123 (73) | 96 (69) | 0.42 | 473 (82) | 464 (84) | 0.50 | 0.008 | <0.001 |
| Mean body mass index (kg/m2) (SD) | 27.7 (5.3) | 27.4 (4.6) | 0.54 | 28.3 (4.2) | 28.0 (3.9) | 0.16 | 0.13 | 0.14 |
| Smoking (%) | 47 (28) | 39 (28) | 0.98 | 155 (27) | 139 (25) | 0.49 | 0.78 | 0.47 |
| Lipid lowering therapy (%) | 74 (44) | 59 (43) | 0.79 | 258 (47) | 248 (46) | 0.90 | 0.59 | 0.46 |
| Antihypertensive therapy (%) | 87 (52) | 83 (60) | 0.2 | 261 (46) | 247 (45) | 0.73 | 0.18 | 0.003 |
| Diabetes (%) | 31 (19) | 25 (18) | 0.90 | 80 (14) | 87 (16) | 0.41 | 0.15 | 0.52 |
| Previous coronary heart disease |  |  |  |  |  |  |  |  |
| Myocardial infarction (%) | 10 (6) | 15 (11) | 0.13 | 87 (15) | 81 (15) | 0.76 | 0.002 | 0.24 |
| Percutaneous coronary intervention (%) | 14 (8) | 15 (11) | 0.46 | 85 (15) | 89 (14) | 0.86 | 0.03 | 0.26 |
| Coronary artery bypass grafting (%) | 3 (2) | 4 (3) | 0.52 | 37 (6) | 38 (7) | 0.79 | 0.02 | 0.08 |
| Previous stroke (%) | 8 (5) | 11 (8) | 0.25 | 27 (5) | 23 (4) | 0.64 | 0.98 | 0.07 |
| Mean LDL-cholesterol (mmol/L) (SD) | 3.0 (1.1) | 3.0 (1.0) | 0.89 | 3.0 (1.1) | 2.9 (1.1) | 0.15 | 0.95 | 0.41 |
| Mean systolic blood pressure (mmHg) (SD) | 146 (25) | 152 (29) | 0.07 | 145 (24) | 147 (23) | 0.39 | 0.60 | 0.02 |
| Mean diastolic blood pressure (mmHg) (SD) | 85 (15) | 87 (16) | 0.18 | 87 (14) | 87 (13) | 0.98 | 0.16 | 0.63 |
| Mean left ventricular ejection fraction (%) (SD) | 52 (11) | 53 (19) | 0.84 | 52 (11) | 52 (12) | 0.43 | 0.95 | 0.45 |
| Acute myocardial infarction (%) | 82 (49) | 68 (50) | 0.88 | 285 (50) | 258 (48) | 0.51 | 0.90 | 0.63 |
| *Hospital-based follow-up: women vs. men  ** Primary care-based follow-up: women vs. men | | | | | | | | |

| **Supplemental table 2. Secondary preventive target achievement for cardiovascular risk factors and medication use in women and men two years after acute myocardial infarction (MI), percutaneous coronary intervention (PCI) or coronary artery bypass grafting (CABG).** | | | | | | | | |
| --- | --- | --- | --- | --- | --- | --- | --- | --- |
| **Target achievement, (%)*** | **Women** | | | **Men** | | |  |  |
|  | Hospital-based follow-up | Primary care-based follow-up | *p* | Hospital-based follow-up | Primary care-based follow-up | *p* | *p*** | *p**** |
|  | n=168 | n=139 |  | n=574 | n=553 |  |  |  |
| Blood pressure | 118 (70) | 69 (50) | <0.001 | 367 (64) | 301 (54) | 0.001 | 0.14 | 0.35 |
| LDL-cholesterol | 124 (74) | 70 (53) | <0.001 | 432 (76) | 331 (61) | <0.001 | 0.58 | 0.07 |
| HbA1c (if diabetes, n=240) | 16 (46) | 12 (55) | 0.52 | 41 (44) | 36 (46) | 0.85 | 0.87 | 0.46 |
| Body mass index | 62 (40) | 44 (32) | 0.36 | 127 (22) | 114 (21) | 0.53 | <0.001 | 0.005 |
| Waist circumference | 24 (15) | 11 (8) | 0.08 | 145 (25) | 117 (21) | 0.11 | 0.004 | <0.001 |
| Lipid lowering therapy | 155 (93) | 125 (91) | 0.37 | 548 (96) | 506 (93) | 0.02 | 0.16 | 0.41 |
| Acetylsalicylic acid | 158 (94) | 126 (91) | 0.26 | 544 (95) | 511 (93) | 0.08 | 0.59 | 0.45 |
| Healthy diet | 35 (21) | 6 (4) | <0.001 | 106 (19) | 42 (8) | <0.001 | 0.50 | 0.19 |
| Non-smoking | 136 (81) | 105 (76) | 0.25 | 464 (81) | 448 (81) | 0.94 | 0.97 | 0.15 |
| Physical activity | 101 (60) | 67 (48) | 0.04 | 360 (63) | 288 (52) | <0.001 | 0.54 | 0.41 |
| * Blood pressure <140/90 mmHg, LDL-cholesterol <2.5 mmol/l (until 2017)/<1.8 mmol/l (2018-20200)/<1.4 mmol/l (2021-), HbA1c<53mmol/l (7%), body mass index <25 kg/m2, waist circumference <80 cm (women)/<94 cm (men), daily use of lipid lowering therapy, daily use of acetylsalisylic acid, Smart Diet score ≥36, non-smoking, physical activity of minimum moderate intensity ≥ 150 min weekly  ** Hospital-based follow-up: women vs. men  *** Primary care-based follow-up: women vs. men | | | | | | | | |
